# RT‐QuIC Detection of Pathological α‐Synuclein in Skin Punches of Patients with Lewy Body Disease

**DOI:** 10.1002/mds.28651

**Published:** 2021-05-18

**Authors:** Angela Mammana, Simone Baiardi, Corinne Quadalti, Marcello Rossi, Vincenzo Donadio, Sabina Capellari, Rocco Liguori, Piero Parchi

**Affiliations:** ^1^ IRCCS, Istituto delle Scienze Neurologiche di Bologna (ISNB) Bologna Italy; ^2^ Department of Experimental Diagnostic and Specialty Medicine (DIMES), University of Bologna Bologna Italy; ^3^ Department of Biomedical and Neuromotor Sciences (DIBINEM) University of Bologna Bologna Italy

**Keywords:** real‐time quaking‐induced conversion, biomarker, CSF, synucleinopathies, prions

## Abstract

**Background:**

Evidence suggests that skin represents a suitable matrix for demonstrating α‐synuclein oligomers as a diagnostic biomarker for Lewy body disease.

**Objective:**

The objective of this study was to evaluate the diagnostic performance of skin α‐syn real‐time quaking‐induced conversion assay in patients with Lewy body disease.

**Methods:**

We analyzed skin punches taken in vitam (n = 69) or postmortem (n = 49) from patients with PD, dementia with Lew bodies (DLB), incidental Lewy body pathology, and neurological controls. Seventy‐nine patients underwent both CSF and skin α‐synuclein real‐time quaking‐induced conversion assay.

**Results:**

Overall, the skin α‐synuclein real‐time quaking‐induced conversion assay distinguished Lewy body disease patients with 94.1% accuracy (sensitivity, 89.2%; specificity, 96.3%). Assay sensitivity reached 94.1% in the 17 Lewy body disease patients analyzed in the cervical region. In patients with both CSF and skin samples, the 2 real‐time quaking‐induced conversion assay protocols yielded similar diagnostic accuracy (skin, 97.5%; CSF, 98.7%).

**Conclusion:**

Skin punch biopsies might represent a valid and convenient alternative to CSF analysis to demonstrate Lew body‐related α‐synuclein deposition in patients with Lewy body disease. © 2021 The Authors. *Movement Disorders* published by Wiley Periodicals LLC on behalf of International Parkinson and Movement Disorder Society

The demonstration of abnormal aggregates of amyloidogenic proteins in accessible biospecimens represents a valuable disease‐specific diagnostic biomarker for neurodegenerative diseases.^1^ Recently, seeding assays exploiting the prion‐like properties of these proteins allowed their ultrasensitive detection in various tissues.^1^ The clinical diagnostic value of these assays has been initially demonstrated using cerebrospinal fluid (CSF).^1^, ^2^, ^3^, ^4^, ^5^, ^6^, ^7^ However, recent studies successfully adapted the assay to tissue sources that might be easier to collect or represent an alternative procedure to lumbar puncture (LP).^1^ In this regard, skin punch biopsies are emerging as a promising matrix to be used as an alternative to CSF in prion diseases and synucleinopathies.^8^, ^9^, ^10^, ^11^, ^12^ However, to date, only 2 studies have examined the α‐synuclein (α‐syn) seeding activity in skin samples from patients with Lewy body disease (LBD) using the real‐time quaking‐induced conversion (RT‐QuIC) assay, and only 1 analyzed skin biopsies.^11^, ^12^

In the present study, we evaluated the diagnostic value of skin α‐syn RT‐QuIC in a sample collection comprising both punches taken postmortem in patients with a definite neuropathological diagnosis and biopsies taken in vitam from patients with a highly probable clinical diagnosis. Moreover, we compared the performance of CSF and skin α‐syn RT‐QuIC in a subgroup of patients.

## 1 Methods

Here we provide only a brief description of methodology (see Supplementary Materials S1 for further details). The local ethics committee, Area Vasta Emilia Centro, approved the study, and participants or the next‐of‐kin gave written informed consent.

### 1.1 Study Populations and Inclusion Criteria

We studied skin punches taken postmortem in a consecutive series of 49 neuropathologically verified cases referred to the neuropathology laboratory at Istituto delle Scienze Neurologiche di Bologna, comprising 47 patients affected by rapidly progressive neurological syndromes of varied etiology (Table S1), of which 7 had incidental LB pathology (Table S2), 1 patient with dementia with Lewy bodies (DLB) and 1 with Parkinson's disease (PD). In addition, we examined skin punch biopsies from 28 patients fulfilling the clinical diagnosis of probable or clinically established PD or probable DLB and an additional control group of 41 patients affected by various neurological disorders (Table S1).

Seventy‐nine patients belonging to both cohorts underwent a diagnostic LP in vitam and had CSF in addition to skin samples available for the RT‐QuIC assay. They included 18 patients with a clinical diagnosis of PD (7) or DLB (11), 1 with a postmortem diagnosis of DLB and 60 patients with various neurological disorders.

### 1.2 Skin Biopsy Procedure and Sample Preparation

The sampling protocol differed between the 2 cohorts. In the postmortem group both cervical and thigh 3‐mm punches were available, except for 2 patients in which only the cervical sample was collected. Instead, samples taken in vitam were from a single site: the cervical region in 23 patients, the leg in 24, and the thigh in 22. A second 3‐mm punch, taken at the same time and from the same site of the first one, was available in 17 (cervical), 21 (leg) and 18 (thigh) patients, respectively.

### 1.3 RT‐QuIC Assay

After optimization (see Results section), we chose the K23Q rec‐α‐synuclein as a substrate based on its established optimal performance,^6^, ^13^ and a final dilution of the skin homogenates (SHs) at 10^−2^ (w/v). We ran the skin RT‐QuIC assay as previously reported^6^ following the protocols applied to brain homogenates and the CSF RT‐QuIC as reported.^7^

### 1.4 Neuropathological Studies

Neuropathological data were collected using standardized procedures and established diagnostic criteria as described.^7^

## 2 Results

### 2.1 Optimization of Experimental Conditions for Skin α‐Synuclein RT‐QuIC

**FIG. 1:**
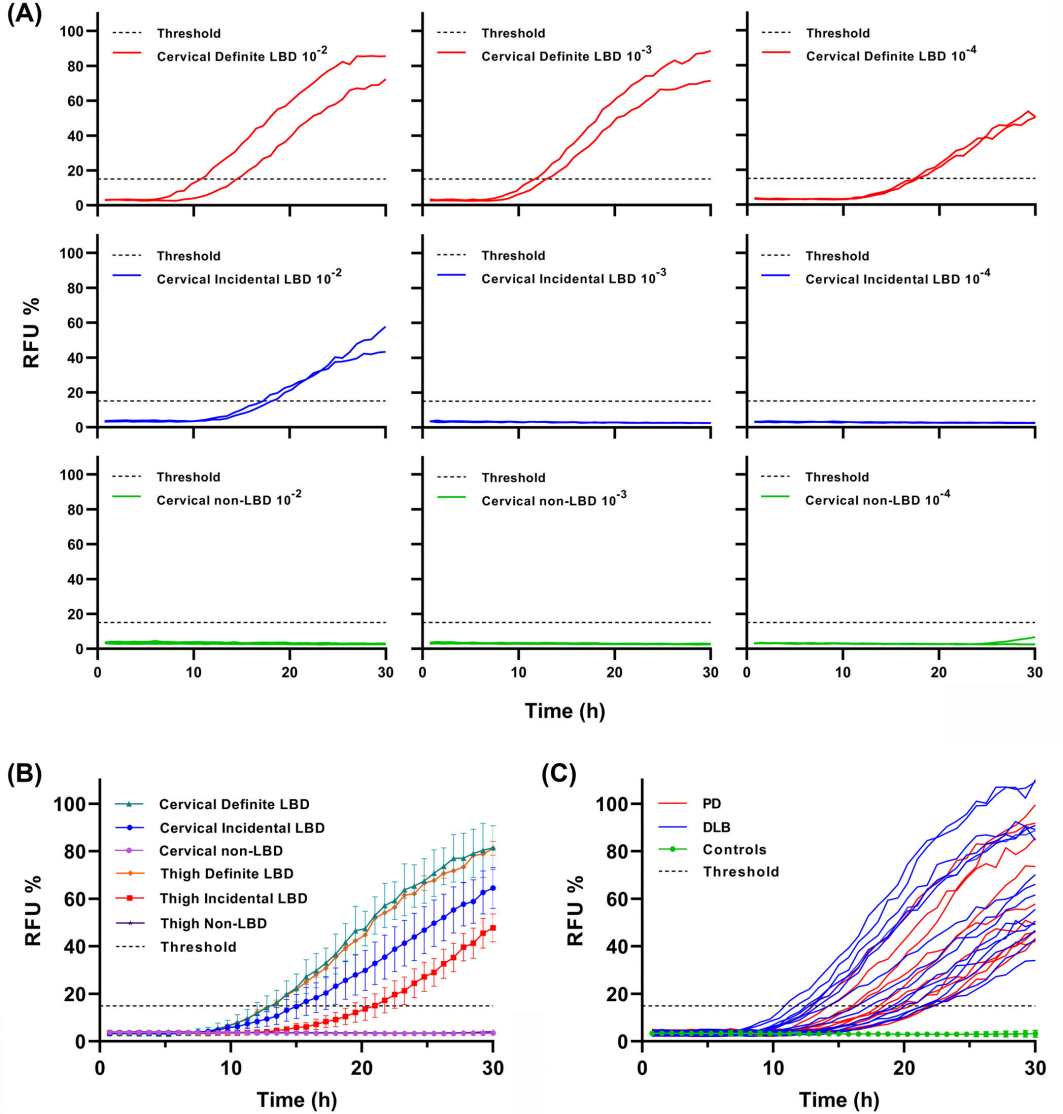
Detection of α‐synuclein seeding activity by skin real‐time quaking‐induced conversion (RT‐QuIC) assay in patients with LBD. (**A**) RT‐QuIC end‐point dilution analysis of LBD and non‐LBD skin homogenates from cervical samples. Serial dilutions (10^−2^, 10^−3^, and 10^−4^) of skin homogenates from 2 definite LBD (top), 2 incidental LBD (middle), and 4 non‐LBD patients (bottom) were tested. Each curve represents the average of the 4 replicates run for each sample. **(B**) Comparison among cervical and thigh skin homogenate seeding activity of neuropathologically confirmed LBD (n = 2 for both sites) and incidental LBD (cervical, n = 7; thigh, n = 6) and non‐LBD (cervical, n = 40; thigh, n = 39) samples. Each curve represents the average of the analyzed group/site. (**C**) RT‐QuIC detection of α‐synuclein seeding activity in skin homogenates from the in vitam cohort patients (results from the first analysis are shown). Each curve depicts the average of quadruplicates run for each LBD patient (PD, n = 13: DLB, n = 15). Non‐LBD patients (n = 41) are represented as the average of the group. All data were normalized to the percentage of the maximal fluorescence response of the positive control included in each experiment. Error bars indicate the standard error of the mean.

We tested various dilutions (w/v) of SHs (from 10^−2^ to 10^−4^) in the RT‐QuIC reaction mixture using cervical samples from the postmortem cohort including 4 LBD patients (1 PD, 1 DLB, and 2 incidental LBD) and 4 non‐LBD controls. Among the tested conditions, only the 10^−2^ (w/v) dilution consistently amplified the fluorescence signal in all LBD samples, giving the most effective reaction in terms of distinction between positive and negative outcomes (Fig. 1A).

### 2.2 RT‐QuIC Detection of α‐Synuclein Seeding Activity in Skin Punches Taken Postmortem

In the chosen experimental conditions, 8 of 9 cervical samples from patients with LB pathology, including 1 patient with only focal LB copathology (Braak LB stage 1), showed a full 4/4 positive response (sensitivity, 88.9%). On the other hand, all but 1 of the non‐LBD cervical samples yielded a negative response (specificity, 97.5%; Fig. 1B, Table 1).

**TABLE 1:** Demographic features of the studied cohorts and results of RT‐QuIC analyses

	In vitam			Postmortem		
	DLB	PD	Controls	DLB, PD	iLBD	Non‐LBD
n	15	13	41	2	7	40
Age at biopsy or death (years)	75.0 ± 7.9	68.2 ± 6.7	66.8 ± 11.4	79, 67	74.6 ± 8.3	71.0 ± 8.7
Time onset to biopsy or autopsy (years)	3.9 ± 2.8	3.3 ± 2.2	—	17.8, 17.0	—	—
α‐syn RT‐QuIC positive/tested (%)						
CSF	11/11 (100)	7/7 (100)	0/27 (0)	1/1 (100)	3/4 (75.0)	0/30 (0)
Skin cervical	4/4 (100)	4/4 (100)	1/15 (6.7)	2/2 (100)	6/7 (85.7)	1/40 (2.5)
Skin thigh	7/7 (100)	2/4 (50.0)	1/11 (9.1)	2/2 (100)	4/6 (66.7)	0/39 (0)
Skin leg	4/4 (100)	4/5 (80.0)	0/15 (0)	—	—	—
Skin overall^a^	15/15 (100)	10/13 (76.9)		2/2 (100)	6/7 (85.7)	
	25/28 (89.3)		2/41 (4.9)	8/9 (88.9)		1/40 (2.5)

Years are expressed as mean ± standard deviation.

^a^ Patients examined postmortem were considered positive when at least 1 of the 2 sites showed α‐syn seeding activity.

CSF, cerebrospinal fluid; DLB, dementia with Lewy bodies; iLBD, incidental Lewy body disease; PD, Parkinson's disease; RT‐QuIC, real‐time quaking‐induced conversion.

Among the 8 patients who tested positive in the cervical site, 7 were also analyzed in the thigh, and 6 of 7 gave a positive response but with fewer positive replicates in some cases (Fig. 1B, Table S2). However, the 2 patients with DLB and PD gave a full 4/4 positive response at both sites. Moreover, all 39 non‐LBD thigh samples showed a negative response. Finally, there were no significant differences in the kinetics of positive replicates between samples from the 2 examined sites (data not shown).

### 2.3 RT‐QuIC Detection of α‐Synuclein Seeding Activity in Skin Punch Biopsies Taken In Vitam

Twenty‐five of the 28 patients with probable LBD showed positive seeding activity (sensitivity, 89.3%), whereas 39 of 41 non‐LBD tested negative (specificity, 95.1%; Fig. 1C, Table 1). Two of the 3 RT‐QuIC‐negative skin samples of the LBD group were from the thigh, the third from the leg. The RT‐QuIC assay with the second biopsy, available for most patients (26 LBD and 30 non‐LBD controls), showed a 98.2% concordance with the first analysis in the positive/negative outcomes, including the negative results in 2 LBD patients not showing α‐syn seeding activity in the first assay. Furthermore, all LBD samples initially showing a positive reaction confirmed the result in the second assay. Regarding the number of positive replicates, all but 2 positive samples gave a complete 4/4 response in both biopsies. Exceptions included 2 samples with 3/4 positive replicates, which in the second analysis gave 2/4 and 4/4. In the DLB group, only 1 specimen (thigh region) showed a positive response (4/4) in the first analysis but no seeding activity in the second one. There were no significant differences in the kinetics of the fluorescence response between PD and DLB patients (data not shown).

### 2.4 Comparison of α‐Synuclein Seeding Activity Detection in Skin and CSF Samples From the Same Patient

CSF samples were available in 79 patients, which allowed us to compare the RT‐QuIC results obtained using both skin and CSF (Table 1). The results showed a very high concordance (76 of 79, 96.2%) between the positive and negative outcomes of the 2 assays. The 3 discordant cases comprised 1 control from the in vitam cohort who was CSF negative but skin positive and 2 LBD patients from the postmortem cohort showing an inverse positive/negative discordance (1 skin positive but CSF negative and 1 skin negative but CSF positive).

In addition, we found no significant differences in the percentage of full (4/4) positive responses between the skin and CSF positive samples in both the postmortem (*P* = 0.24) and the in vitam (*P* > 0.99) cohorts.

## 3 Discussion

In this study we have demonstrated that the RT‐QuIC efficiently detects abnormal α‐syn seeds in skin punches from patients with PD, DLB, and incidental LB pathology. Overall, skin α‐syn RT‐QuIC showed 94.1% accuracy (sensitivity, 89.2%; specificity, 96.3%; 95% CI, 88.2%–97.6%) in distinguishing LBD patients from neurological controls in the whole cohort and 92.8% accuracy (sensitivity, 89.3%; specificity, 95.1%; 95% CI, 83.9%–97.6%) in those tested in vitam. However, the higher positivity rate we obtained with cervical punches suggests that the assay accuracy could have been even higher if we had the cervical punch available in all patients. Similarly, our findings of positive α‐syn seeds in patients with incidental LB pathology limited to a few regions in the brainstem suggest that positive RT‐QuIC reactions in elderly clinical controls could represent a “genuine” seeding activity at a presymptomatic disease stage rather than a “false‐positive” result. In this respect, it is noteworthy that one of our potential false‐positive samples belonged to a patient with probable mild cognitive impairment–Alzheimer's disease, a condition with a relatively high incidence of LBD copathology.

The present results are in line with previous immunohistochemical (IHC) studies in LBD patients demonstrating the accumulation of α‐syn in peripheral organs, including skin, salivary gland, and gut.^14^, ^15^, ^16^, ^17^ Among them, the skin showed the highest value as a diagnostic biomarker in patients with PD and DLB. However, cutaneous misfolded α‐syn detection by IHC suffers from limitations related primarily to the patchy availability of autonomic nerves in a given section, which lowers its sensitivity unless multiple samples are analyzed. Furthermore, the methods used in the most promising studies required specialized and laborious sample processing and slide interpretation.^18^, ^19^

Regarding the most suitable anatomical site for the skin analysis, our data, although preliminary in this respect, seems to confirm the higher value of the cervical area.^19^ Indeed, all 3 patients with clinical PD diagnosis, which gave a negative RT‐QuIC response, were analyzed in distal locations (thigh or leg). Moreover, in the postmorten cohort, the RT‐QuIC showed a positive response limited to the cervical site in 1 patient with incidental LB pathology. Finally, we also detected a trend toward higher fluorescence reactivity in cervical samples compared with the thigh site.

In this study, we also directly compared the performance of skin and CSF α‐syn RT‐QuIC. The results showed very high concordance between the 2 protocols with only slight differences in sensitivity and specificity, mainly attributable to the small number of samples available for this subanalysis. The agreement, especially regarding sensitivity, is also demonstrated by the positive α‐syn seeding activity shown by patients with incidental LBD and a relatively low Braak stage^20^ of LB pathology (≤3) using both approaches. Of note, compared with PD and DLB samples, those from the incidental LBD group gave an earlier negative response in more diluted samples, suggesting lower α‐syn seeding activity reflecting the focal CNS LB pathology in these samples. Further studies should explore the sample dilution curve responses as a possibility to improve the value of the RT‐QuIC as a quantitative assay of α‐syn seeding activity.

Overall, the present data, combined with those of 2 previous studies,^11^, ^12^ indicate that skin α‐syn RT‐QuIC may represent an assay with comparable diagnostic value to cutaneous IHC but less technical limitations. The findings support its use, either in combination or as an alternative to CSF analysis, in patients with contraindications to LP. Skin punch biopsy might also be preferable to LP, given its low invasiveness, for the future screening of patients with prodromal syndromes that are significantly associated with LB pathology.^7^, ^21^

In conclusion, our study supports the use of skin punch biopsies as an alternative or in support of CSF analysis to demonstrate α‐syn seeding activity in patients with PD, DLB, and, possibly, incidental LB pathology in vitam.

## Author Roles

A.M., S.B., and P.P. contributed to the conception and design of the study. V.D, performed the skin biopsies. S.B. and P.P. took the skin samples at autopsy and performed the neuropathological analysis. A.M. performed the RT‐QuIC assays. A.M. and C.Q. analyzed the RT‐QuIC data. M.R. and C.Q. prepared the recombinant synuclein substrate. S.B, S.C., V.D., R.L., and P.P contributed to the acquisition and/or analysis of clinical data. A.M., C.Q., and P.P. contributed to drafting the text and preparing the figure. P.P. supervised the study. All authors reviewed the article for intellectual content.

## Full Financial Disclosure for the Previous 12 Months

R.L. received lecture fees from LT3, PTS, and Galen Symposion and advisory board consultancy fees from Argenx BV, Alexion Pharma‐Italy, and PREX. None of the other authors have financial disclosure to report.

## Supporting information


**Appendix S1.** Supporting Information

## Data Availability

The data sets generated and/or analyzed during the current study are available from the corresponding author on reasonable request.
