# Supplementary material for: RT‐QuIC Detection of Pathological α‐Synuclein in Skin Punches of Patients with Lewy Body Disease
Source: Mov Disord. 2021 May 18;36(9):2173–7. doi: 10.1002/mds.28651 (PMC8518528; doi:10.1002/mds.28651)
Supplement: Supplementary file 1 — Appendix S1. Supporting Information [file MDS-36-2173-s001.docx]

**RT-QuIC detection of pathological α-synuclein in skin punches of patients with Lewy body disease**

Angela Mammana, MSc^1^, Simone Baiardi MD, PhD^1,2^, Corinne Quadalti PhD^1^, Marcello Rossi MSc^1^, Vincenzo Donadio MD^1^, Sabina Capellari MD^1,3^, Rocco Liguori MD^1,3^, Piero Parchi MD, PhD^1,2*^.

**Supplementary material S1.**

**Patient classification**

All patients were referred to the Institute of Neurological Science of Bologna (ISNB), Italy, between 2018 and 2020. Patients with LBD were diagnosed according to Movement Disorder Society Clinical Diagnostic Criteria for PD and DLB Consortium Criteria,^1,2^ based on the agreement of two consultant neurologists who independently reviewed the medical charts and formulated the final diagnosis before knowing the result of the RT-QuIC assay.

**Skin biopsy procedure and sample preparation**

Skin tissues were collected using a 3 mm punch at the cervical C7 paravertebral area or the lateral surface of the thigh (~20 cm above the patella) or 10 cm proximal to the lateral malleolus and kept frozen at -80°C until use.

After thawing, tissue was washed 3 times in cold 1X PBS and processed as previously described ^3^ except for the use of 1X PBS as homogenization and dilution buffer.

**RT-QuIC assay.**

The following variables were used to quantitate the RT-QuIC output: the time taken by the signal to reach the threshold (Lag phase), the maximum fluorescence read (Imax) and the area under the curve (AUC). The RT-QuIC reaction was deemed to be positive if at least two out of the four replicates gave a fluorescence signal higher than the chosen threshold cut-off value. The latter was set as the 15% of the maximum value reached by any of the positive control replicates during the 30-hour run. The choice of the 15% value was based on the analysis of the variability of fluorescence signal in a group (n=20) of non-LBD negative controls (mainly patients with a clinical diagnosis of ALS or peripheral neuropathy)*.* The threshold set at 15% indicates the lowest value granting clear discrimination between positive and negative replicates. The value corresponded to the mean of the signal of negative controls plus approximately 10 SD. The threshold is calculated separately for each 96-well plate to also limit the possible inter-experimental variability related to the use of different plate readers.^4^. As positive controls, we used a skin sample from the patient with a definite post-mortem diagnosis of DLB included in the study, and a pool of CSF samples from a previous study, showing a complete 4/4 positive response.

Samples giving a positive signal in a single replicate were classified as “unclear,” repeated up to three times, and eventually deemed negative unless giving a definite positive response (i.e., at least 2/4). All RT-QuIC experiments and data analysis were performed by personnel blinded to the clinical diagnostic groups.

**Neuropathological studies**

To assess neurodegenerative pathologies, immunohistochemistry with antibodies specific for α-syn (LB509, dilution 1:100, Thermo Fisher Scientific, and KM51, dilution 1:500, Novocastra), p-tau (AT8, dilution 1:100, Innogenetics), Aβ (4G8, dilution 1:5000, Signet Labs) and prion protein (3F4, dilution 1:400, Signet Labs) was applied to all cases using several brain regions according to established consensus criteria as described.^5,6^

**Statistical analyses**

RT-QuIC relative fluorescence responses were analysed and plotted using GraphPad Prism 8.4.0 for Windows. The Mann-Whitney test was used to reveal differences between two groups; the Fisher’s exact test was used to compare the proportion of 4/4 positive replicates between skin and CSF RT-QuIC outcomes; the unpaired Student T test was used to compare RT-QuIC quantitative values between two groups; p value <0.05 was considered statistically significant. Unless stated otherwise, data are expressed as mean with standard error of the mean (SEM).

**Detailed study of discordant cases from the ex vivo group.**

**Patient a.** RT-QuIC analysis of skin samples of case #9 (see Table S2) from both the cervical and thigh sites gave a negative response. In contrast, the CSF sample collected in-vitam showed a positive signal (2/4 positive wells). Her medical history was relevant for rapidly progressive cognitive decline started about one year before death, memory loss, behavioral change, and falls. The patient also had two ischemic strokes and developed post-stroke seizures. Neuropathological examination showed a large ischemic stroke involving the parietal and occipital regions of the left hemisphere. Small vessel disease was evident in the subcortical white matter, amygdala, basal ganglia, and thalamus. Diffuse and focal beta-amyloid deposits were evident in the neocortices, hippocampus, amygdala, striatum, and periaqueductal gray (Thal phase 4). Immunohistochemistry for p-tau revealed abnormal deposits in the entorhinal cortex, hippocampus, and parahippocampal gyrus (Braak stage of neurofibrillary pathology III). Lewy body pathology involved the medulla oblongata, pons, and substantia nigra (Braak stage of Lewy body pathology 3).

**Patient b**. A 61-year old patient (case #6 in Table S2) with a history of rapidly progressive dementia (total disease duration 6 months) and diagnostic investigations (EEG, MRI) consistent with probable Creutzfeldt-Jakob disease underwent autopsy 6-month after symptom onset. CSF was not available for analysis. Neuropathological examination confirmed the diagnosis of sporadic Creutzfeldt-Jakob disease. No Lewy body pathology was detected in the left encephalus (the other half was frozen), even after the analysis of serial brainstem sections. The olfactory bulb and the spinal cord were not available. The a-syn RT-QuIC assay gave a positive result in the cervical site (4/4 positive wells) but a negative response (0/4) in the thigh.

**Patient c**. A 62-year old female (Case #7 in Table S2) with a clinical diagnosis of probable Creutzfeldt-Jakob disease underwent autopsy 8-month after the disease onset. CSF collected in vitam was tested negative by α-syn RT-QuIC. Neuropathological examination confirmed the diagnosis of sporadic Creutzfeldt-Jakob disease. Lewy bodies and neurites were detected in the medulla oblongata, pons, and substantia nigra (Braak stage of Lewy body pathology 3). The a-syn RT-QuIC assay gave a positive result in both the cervical site (4/4 positive wells) and the thigh (2/4).

**Table S1.** List of diagnoses in neuropathological and clinical non-LB cohorts.

| **Diagnostic categories** | **N** | **Age at skin sample collection, years** |
| --- | --- | --- |
| **Neuropathological non-LB group**  **(primary diagnosis)** | | |
| Sporadic Creutzfeldt-Jakob disease | 25 | 70.9±9.2 |
| Genetic Creutzfeldt-Jakob disease | 2 | 74.0±7.0 |
| Alzheimer’s disease | 2 | 75.5±0.7 |
| Wernicke encephalopathy | 2 | 67.5±2.1 |
| Autoimmune encephalitis | 1 | 85 |
| Hypoxic encephalopathy | 1 | 66 |
| Subcortical vascular encephalopathy | 1 | 73 |
| Mixed dementia | 1 | 81 |
| Intracerebral hemorrhage | 1 | 54 |
| Non-specific neuropathological change | 4 | 68.3±6.7 |
| **Clinical non-LB cohort** | | |
| Peripheral neuropathy | 12 | 63.1±13.0 |
| Alzheimer’s disease | 9 | 71.8±10.4 |
| Vascular dementia | 5 | 71.2±9.0 |
| Motor neuron disease | 4 | 59.8±7.3 |
| Frontotemporal dementia | 4 | 74.3±7.6 |
| Subjective cognitive decline | 2 | 51.5±3.5 |
| Corticobasal syndrome | 1 | 77 |
| Autoimmune encephalitis | 1 | 66 |
| Gerstmann-Sträussler-Scheinker disease | 1 | 41 |
| Stiff-person syndrome | 1 | 75 |
| Fragile X syndrome | 1 | 63 |

Age is expressed as mean±standard deviation. LB Lewy bodies.

**Table S2.** Neuropathological features and results of the α-Syn RT-QuIC assay of the definite and incidental LBD group

| List of cases | Sex | Age at death | Primary NP diagnosis | Secondary NP diagnosis | Braak LB stage | RT-QuIC result^b^ | |
| --- | --- | --- | --- | --- | --- | --- | --- |
|  |  |  |  |  |  | Cervical | Thigh |
| Case #1 | F | 79 | DLB | AD^c^ (intermediate) | 6 | 4/4 | 4/4 |
| Case #2 | M | 67 | PD | AD^c^ (mild) | 5 | 4/4 | 4/4 |
| Case #3 | M | 76 | VaE, CAA, AD^c^ (mild) | ILB pathology | 4 | 4/4 | 2/4 |
| Case #4^a^ | M | 76 | sCJD MM1+2C | ILB pathology, AGD | 3 | 4/4 | 3/4 |
| Case #5 | F | 82 | AD^c^ (intermediate) | ILB pathology, hippocampal sclerosis, LATE-NC, ARTAG | 1 | 3/4 | 3/4 |
| Case #6^a^ | F | 64 | sCJD MM1+2C | ILB pathology | 3 | 4/4 | 0/4 |
| Case #7^a^ | F | 62 | sCJD MM1 | ILB pathology | 3 | 4/4 | 2/4 |
| Case #8 | F | 82 | Primary CNS lymphoma | ILB pathology, PART | 4 | 4/4 | NA |
| Case #9 | F | 80 | Cerebral ischemic stroke | Subcortical VaE, AD^c^ (intermediate), ILB pathology | 3 | 0/4 | 0/4 |

NP, neuropathologic; LB, Lewy bodies; RT-QuIC, real-time quaking-induced conversion; DLB, dementia with Lewy bodies; AD, Alzheimer’s disease; PD, Parkinson’s disease; VaE, Vascular Encephalopathy; CAA, cerebral amyloid angiopathy; ILB, incidental Lewy body; sCJD, sporadic Creutzfeldt-Jakob disease; AGD, argyrophilic grain disease; LATE-NC, Limbic-predominant age-related TDP-43 encephalopathy neuropathological change; ARTAG, aging-related tau astrogliopathy; CNS, central nervous system; PART, primary age-related tauopathy.

^a^sCJD subtypes were defined in accordance with Parchi et al.^7^

^b^ Number of positive replicates out of total tested replicates.

^c^ The severity of AD neuropathologic change was assessed according to Montine et al. 2012.^8^

**Supplementary references.**

1. Postuma RB, Berg D, Stern M, Poewe W, Olanow CW, Oertel W et al (2015) MDS clinical diagnostic criteria for Parkinson’s disease. Mov Disord 30(12):1591–1601.

2. McKeith IG, Boeve BF, Dickson DW, et al. Diagnosis and management of dementia with Lewy bodies: fourth consensus report of the DLB Consortium. Neurology 2017;89(1):88–100.

3. Mammana A, Baiardi S, Rossi M, et al. Detection of prions in skin punch biopsies of Creutzfeldt-Jakob disease patients. Ann Clin Transl Neurol 2020;7(4):559-564.

4. Orrù CD, Ma TC, Hughson AG, et al. A rapid α-synuclein seed assay of Parkinson's disease CSF panel shows high diagnostic accuracy. Ann Clin Transl Neurol 2021;8(2):374-384.

5. Alafuzoff I, Arzberger T, Al-Sarraj S, et al. Staging of neurofibrillary pathology in Alzheimer's disease: a study of the BrainNet Europe Consortium. Brain Pathol 2008;18(4):484–496.

6. Alafuzoff I, Ince PG, Arzberger T, et al. Staging/typing of Lewy body related alpha-synuclein pathology: a study of the BrainNet Europe Consortium. Acta Neuropathol 2009;117(6):635–652.

7. Parchi P, de Boni L, Saverioni D, et al. Consensus classification of human prion disease histotypes allows reliable identification of molecular subtypes: an inter-rater study among surveillance centres in Europe and USA. Acta Neuropathol 2012;124(4):517–529.

8. Montine TJ, Phelps CH, Beach TG, et al. National Institute on Aging-Alzheimer's Association guidelines for the neuropathologic assessment of Alzheimer's disease: a practical approach. Acta Neuropathol 2012;123(1):1–11.
